# Hydroxyl Radical Formation: A Key Mechanism and Regulatory Target in Monascin Photo-Degradation

**DOI:** 10.3390/jof12080561

**Published:** 2026-07-31

**Authors:** Zhihan Shang, Xiaowei Zhang, Mingfei Li, Qian Lu, Fenghe Wang, Ting Chen, Yang Li, Yizheng Liu

**Affiliations:** 1 School of Grain Science and Technology, Jiangsu University of Science and Technology, Zhenjiang 212100, China; xingxiangzi_lanyu@163.com (Z.S.); limingfei@just.edu.cn (M.L.); luqian@just.edu.cn (Q.L.); fhwang_056@163.com (F.W.); chenzhuqi_77@163.com (T.C.); yangli@just.edu.cn (Y.L.); 15653365403@163.com (Y.L.); 2 Jiangsu Provincial Engineering Research Center of Grain Bioprocessing, Zhenjiang 212100, China

**Keywords:** Monascin, photo-degradation, hydroxyl radicals, dissolved oxygen, antioxidants

## Abstract

Monascin, a yellow azaphilone pigment derived from *Monascus* fermentation, undergoes significant photo-degradation that limits its applications. This study reveals that free radical formation, particularly hydroxyl radicals (·OH) generated via H_2_O_2_ photolysis, is the primary mechanism driving monascin photo-degradation. Dissolved oxygen synergistically accelerates degradation, whereas deoxygenation reduces radical yield by 68% and lowers the degradation to 5%. Enzymatic scavengers confirmed H_2_O_2_ as the critical ·OH precursor: catalase reduced radicals by 61% and suppressed degradation to 7%, while superoxide dismutase exhibited moderate effects. Natural antioxidants ascorbic acid and α-lipoic acid (ALA) protected monascin concentration-dependently, with ALA showing superior photo-protection (68% degradation reduction) due to its broad ROS-scavenging capacity. These findings establish H_2_O_2_-derived ·OH as the primary destructive species and identify oxygen exclusion, H_2_O_2_ decomposition, and radical scavenging as effective photo-stabilization strategies.

## 1. Introduction

*Monascus* pigments, a class of secondary metabolites produced by fermentation of *Monascus* species, have been widely used as natural food colorants for centuries, particularly in Asian cuisine [1,2]. Among these pigments, monascin, a yellow azaphilone compound, has attracted considerable attention not only for its coloring properties but also for its reported biological activities, including anti-inflammatory, antioxidative, and anti-diabetic effects [1,3,4]. These multifunctional attributes position monascin as a promising natural additive for functional food development.

Despite these advantageous attributes, the practical application of monascin is severely constrained by its susceptibility to photo-degradation. Upon exposure to light, particularly in the ultraviolet and visible regions, monascin undergoes rapid structural degradation accompanied by color fading, which compromises both product quality and consumer acceptance [5,6]. Elucidating the fundamental mechanisms underlying this photo-degradation process is therefore essential for developing effective stabilization strategies.

Previous studies have suggested that the photo-degradation of azaphilone pigments may involve reactive oxygen species (ROS)-mediated pathways. Light absorption by pigment molecules can promote transition to excited states, facilitating electron transfer and subsequent generation of free radicals [7,8]. Among various ROS, hydroxyl radicals (·OH) are particularly destructive due to their high oxidation potential and ability to attack conjugated chromophores, leading to ring opening and irreversible bleaching [9,10]. Hydrogen peroxide (H_2_O_2_) and superoxide anions (O_2_^−^·) have been implicated as intermediate species in such radical cascades, with H_2_O_2_ serving as a direct precursor to ·OH via photolytic cleavage or Fenton chemistry [11,12].

Environmental factors, especially molecular oxygen, play a critical role in photo-sensitized oxidation reactions. Oxygen can interact with excited triplet-state chromophores to generate ROS, amplifying oxidative damage to pigment structures [13,14,15]. Consequently, strategies that limit oxygen availability or intercept ROS have been explored to enhance pigment photo-stability. Natural antioxidants, including ascorbic acid and α-lipoic acid, are known to scavenge various ROS and have been applied to protect labile compounds from oxidative degradation [16,17,18]. However, their efficacy in protecting monascin against light-induced degradation has not been systematically evaluated.

Despite the recognized susceptibility of monascin to photo-degradation, the precise molecular mechanisms remain incompletely understood. First, direct evidence for free radical generation during monascin photo-degradation has yet to be established. Second, the specific contributions of individual ROS (H_2_O_2_, O_2_^−^·, and ·OH) to the overall degradation process and their interrelationships remain unclear. Third, the relative effectiveness of targeted intervention strategies, including oxygen exclusion, enzymatic ROS scavenging, and antioxidant protection, has not been systematically compared. Addressing these gaps is a prerequisite for rational design of photo-stabilization approaches in food applications.

On the basis of the above considerations, we hypothesize that ·OH, generated via photolytic cleavage of monascin, constitutes the primary reactive species responsible for monascin photo-degradation. We further propose that the extent of photo-degradation is directly modulated by the availability of dissolved oxygen and the presence of radical scavengers, and that targeted elimination of ·OH, through deoxygenation or enzymatic or antioxidant interception, can effectively protect monascin from light-induced degradation.

Therefore, this study aimed (1) investigate the generation of free radicals during monascin photo-degradation using electron spin resonance (ESR) spectroscopy coupled with spin-trapping, (2) elucidate the roles of dissolved oxygen and specific ROS (H_2_O_2_ and O_2_^−^·) through deoxygenation experiments and enzyme-based scavenging studies with catalase (CAT) and superoxide dismutase (SOD), (3) evaluate the photo-protective effects of two representative natural antioxidants, ascorbic acid and α-lipoic acid, (4) identify key regulatory targets for enhancing monascin photo-stability. The findings are expected to provide a mechanistic foundation for developing practical strategies to improve the light stability of monascin in food and nutraceutical systems.

## 2. Materials and Methods

### 2.1. Materials and Reagents

Monascin (purity ≥ 98%) was purchased from Sigma-Aldrich (St. Louis, MO, USA), as well as hydrogen peroxide (H_2_O_2_, 30%) and acetonitrile (HPLC-grade). 5-tert-Butoxycarbonyl-5-methyl-1-pyrroline-N-oxide (BMPO) was obtained from Dojindo Laboratories (Kumamoto, Japan) and used as the spin-trapping agent for electron spin resonance (ESR) spectroscopy. Catalase (CAT, from bovine liver, ≥2000 U/mg protein), superoxide dismutase (SOD, from bovine erythrocytes, ≥2500 U/mg protein), ascorbic acid (AA, ≥99%), and α-lipoic acid (ALA, ≥99%) were purchased from Sigma-Aldrich. All other chemicals and reagents were of analytical grade. A UV-Vis Spectrophotometer (UV9600, Shanghai Shuangxu Electronics Co., Ltd., Shanghai, China), xenon light source (HF-GHX-XE-300, Shanghai Hefan Instrument Co., Ltd., Shanghai, China), and radiometer (FZ-A; Shanghai Hefan Instrument Co., Ltd., Shanghai, China) were used in this study.

### 2.2. Effect of Light on Monascin Stability

Monascin is an azaphilone compound possessing a conjugated double-bond system, which confers a characteristic absorption maximum at 385 nm in 70% acetonitrile, as determined by UV-Vis spectrophotometry. The absorbance at this wavelength is directly proportional to monascin concentration, and the concentration change can be evaluated by tracking the decrease in absorbance at 385 nm. The photo-stability of different concentrations of monascin was evaluated under simulated solar light irradiation. A stock solution of 0.3 mM monascin was prepared in 70% acetonitrile. Working solutions with concentrations of 0.06, 0.12, 0.18, and 0.24 mM were obtained by appropriate dilution of the stock. For each concentration, 24 mL of solution was dispensed into eight stoppered quartz cuvettes (3 mL per tube, 10 mm path length). The cuvettes were randomly divided into two groups, one for light irradiation and the other for dark controls. The light group was placed 15 cm from a xenon lamp equipped with an AM 1.5 G filter, which provided simulated solar light (290–800 nm) at an irradiance of 100 mW/cm^2^, as measured by an optical power meter. The dark controls were wrapped in aluminum foil and kept under identical conditions. Both groups were simultaneously exposed for 20 min. At 5 min intervals (5, 10, 15, and 20 min), one cuvette from each group was removed, and the absorbance at 385 nm was immediately recorded. To minimize errors caused by solvent evaporation during irradiation, the volume of each sample was adjusted to the 3.0 mL mark with 70% acetonitrile immediately before and after each irradiation period, ensuring a constant volume for absorbance measurements. The degradation rate was calculated according to Formula (1).

(1) $$R=(1-\frac{A_{1}}{A_{0}})\times 100\%$$

R is the degradation rate of monascin.

A1 is the absorbance of monascin at 385 nm after illumination for a certain time.

A0 is the absorbance of monascin at 385 nm before illumination.

### 2.3. Effect of H_2_O_2_ on Monascin Stability

To evaluate the direct oxidative effect of hydrogen peroxide on monascin in the absence of light, a stock solution of 0.3 mM monascin was prepared in 70% acetonitrile. A total of 54 mL of this solution was distributed into 20 stoppered quartz cuvettes (2.7 mL per cuvette). The cuvettes were randomly divided into five groups. Four groups received 300 μL of H_2_O_2_ solutions at concentrations of 2.5, 5.0, 7.5, and 10.0 mM, respectively, while the control group received 300 μL of deionized water. The final volume in each cuvette was 3.0 mL, yielding final H_2_O_2_ concentrations of 0.25, 0.50, 0.75, and 1.00 mM for the treatment groups, and 0 mM for the control. All cuvettes were incubated in the dark for 20 min. At each time point (5, 10, 15, and 20 min), one cuvette from each group was removed, and the absorbance at 385 nm was immediately recorded. The degradation rate was calculated according to Formula (1). All experiments were performed in triplicate.

### 2.4. Combined Effect of Light and H_2_O_2_ on Monascin Stability

The synergistic effect of simulated solar light and H_2_O_2_ on monascin degradation was investigated using the same sample preparation as that described in Section 2.3. Briefly, five groups of samples were prepared and irradiated under simulated solar light at an irradiance of 100 mW/cm^2^ for 20 min, following the conditions described in Section 2.2. At 5 min intervals (5, 10, 15, and 20 min), one tube from each group was removed, and the absorbance at 385 nm was immediately recorded. The degradation rate was calculated according to Formula (1). All experiments were performed in triplicate.

### 2.5. Effect of Dissolved Oxygen on the Photodegradation of Monascin

To evaluate the influence of deoxygenation on monascin photo-stability, 24 mL of 0.3 mM monascin solution in 70% acetonitrile was evenly distributed into eight stoppered quartz colorimetric tubes (10 mL capacity, 3 mL per tube). The tubes were randomly divided into two groups of four tubes each. For the deoxygenated group, each tube was purged with high-purity nitrogen gas (99.999%) at a flow rate of 50 mL/min for 30 min to remove dissolved oxygen. For the oxygen-containing group (control), each tube was bubbled with ambient air under the same flow rate and duration [19]. After the 30 min gas purging, all eight tubes were simultaneously placed under simulated solar light at an irradiance of 100 mW/cm^2^ and continuously irradiated for 20 min while gas purging was maintained throughout the irradiation period. At 5 min intervals (5, 10, 15, and 20 min), one tube from each group was removed and immediately sealed, and the absorbance at 385 nm was recorded. The degradation rate was calculated using Formula (1). All experiments were carried out in triplicate.

### 2.6. Effect of CAT and SOD on Monascin Photo-Stability

To elucidate the specific contributions of H_2_O_2_ or superoxide anions (O_2_·^−^) generated during irradiation of monascin solution to its photo-degradation, we evaluated the effects of catalase (CAT), which specifically decomposes H_2_O_2_ into H_2_O and O_2_, and superoxide dismutase (SOD), which catalyzes the dismutation of O_2_·^−^ to H_2_O_2_ and O_2_. These enzymes serve as selective probes to distinguish whether the observed photo-degradation is primarily mediated by H_2_O_2_-derived ·OH or by superoxide-dependent pathways. We anticipated that if H_2_O_2_-derived ·OH is the primary destructive species, CAT would provide substantially greater protection than SOD, even at lower concentrations. A total of 32.4 mL of 0.3 mM monascin solution in 70% acetonitrile was evenly distributed into twelve stoppered quartz colorimetric tubes (10 mL capacity, 2.7 mL per tube). The tubes were randomly divided into three groups of four tubes each. To the first group, 300 μL of CAT solution (100 U/mL) was added to each tube, yielding a final CAT concentration of 10 U/mL. To the second group, 300 μL of SOD solution (1500 U/mL) was added to each tube, yielding a final SOD concentration of 150 U/mL. The third group received 300 μL of deionized water as the control. The final volume in each tube was 3.0 mL. All 12 tubes were placed under simulated solar light and irradiated for 20 min at an irradiance of 100 mW/cm^2^, following the conditions described in Section 2.2. At 5 min intervals (5, 10, 15, and 20 min), one tube from each group was removed, and the absorbance at 385 nm was immediately recorded. The degradation rate was calculated according to Formula (1). All experiments were performed in triplicate.

### 2.7. Effect of Ascorbic Acid and α-Lipoic Acid on Monascin Photo-Stability

The protective effects of ascorbic acid (AA) and α-lipoic acid (ALA) on the photo-stability of monascin were investigated. A total of 75.6 mL of 0.3 mM monascin solution in 70% acetonitrile was evenly distributed into 28 stoppered quartz colorimetric tubes (10 mL capacity, 2.7 mL per tube). The tubes were randomly divided into seven groups of four tubes each. The first group received 300 μL of deionized water as the control. The second, third and fourth groups received 300 μL of AA solutions (containing 0.01 mM EDTA) at concentrations of 10, 30 and 50 mg/mL, respectively, yielding final AA concentrations of 1, 3 and 5 mg/mL. The fifth, sixth and seventh groups received 300 μL of ALA solutions at concentrations of 10, 30 and 50 mg/mL, respectively, yielding final ALA concentrations of 1, 3 and 5 mg/mL. The final volume in each tube was adjusted to 3.0 mL. All tubes were irradiated under simulated solar light for 60 min at 100 mW/cm^2^. At each time point (15, 30, 45, and 60 min), one cuvette from each group was removed, and the absorbance at 385 nm was immediately recorded. The degradation rate was calculated according to Formula (1). All experiments were performed in triplicate.

### 2.8. ESR Spectroscopy for Free Radical Detection

Free radical generation during photo-degradation was detected using a Bruker EMX ESR spectrometer (Billerica, MA, USA) equipped with a high-sensitivity cavity [20]. BMPO was dissolved in ultrapure deionized water (18.2 MΩ·cm) to prepare a 250 mM solution and used as the spin-trapping agent at a final concentration of 25 mM. For each measurement, 40 μL of sample solution, prepared as described in Section 2.2, Section 2.3, Section 2.4, Section 2.5, Section 2.6 and Section 2.7, was mixed with 5 μL of 250 mM BMPO in a glass capillary tube and sealed. For irradiated samples, the capillary tubes were positioned within the ESR cavity and irradiated online with simulated solar light (100 mW/cm^2^) directly through the cavity window for 10 min. ESR spectra were recorded immediately after irradiation. Dark controls were treated identically but without light. All ESR spectra were recorded at room temperature immediately after the designated treatment. The instrument parameters were set as follows: microwave frequency 9.85 GHz, microwave power 20 mW, modulation frequency 100 kHz, modulation amplitude 1.0 G, and scan range 100 G. Signal intensities were quantified by measuring the peak-to-peak height of the second line of the BMPO/·OH adduct spectrum. All measurements were performed in triplicate.

For H_2_O_2_ photolysis experiments, 40 μL of H_2_O_2_ solutions at concentrations of 0.25, 0.50, 0.75, and 1.00 mM in 70% acetonitrile was mixed with 5 μL of 250 mM BMPO in a glass capillary tube and sealed. The capillary tubes were positioned within the ESR cavity and irradiated online with simulated solar light (100 mW/cm^2^) directly through the cavity window for 10 min. ESR spectra were recorded immediately after irradiation under the same instrument conditions described above.

### 2.9. Statistical Analysis

All experiments were performed in triplicate (*n* = 3), and data were expressed as mean ± standard deviation (SD). Statistical analysis was performed using SPSS software version 23.0. Comparisons between groups were conducted using one-way analysis of variance (ANOVA) followed by Tukey’s post hoc test. Differences were considered statistically significant at *p* < 0.05.

## 3. Results and Discussion

### 3.1. Effect of Light and H_2_O_2_ on Monascin Stability

**Figure 1:**
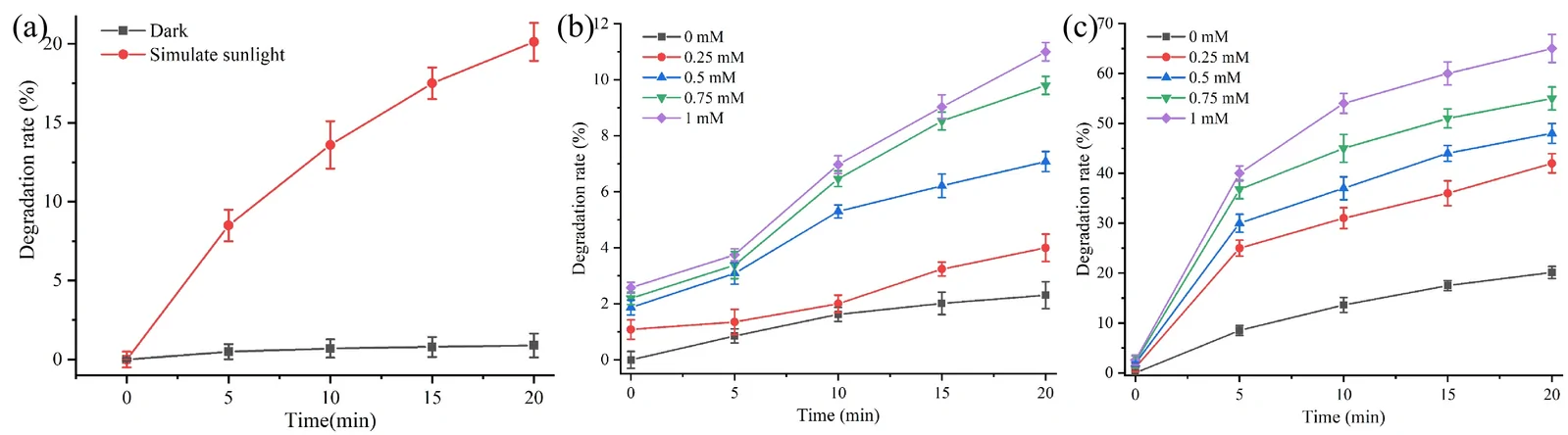
Effect of light and H_2_O_2_ on monascin stability. (**a**) Photo-stability of 0.3 mM monascin under 100 mW/cm^2^ simulated solar light. (**b**) Effect of H_2_O_2_ concentration on monascin stability in the dark. (**c**) Combined effect of light and H_2_O_2_ on monascin stability. Data are presented as mean ± SD (*n* = 3).

The stability of 0.3 mM monascin was investigated under 100 mW/cm^2^ simulated solar light alone, treatment with different H_2_O_2_ concentrations (0.25 mM, 0.5 mM, 0.75 mM, 1 mM), and the combination of light and H_2_O_2_ (Figure 1).

As shown in Figure 1a, irradiation with simulated solar light for 20 min resulted in a 20.1% degradation of monascin, confirming that monascin is intrinsically photosensitive. In the dark, treatment with 0.25–1.0 mM H_2_O_2_ for the same duration produced only up to 11% degradation (Figure 1b), indicating that direct oxidation by H_2_O_2_ alone is relatively mild under these conditions. However, when light irradiation was combined with H_2_O_2_, degradation increased dramatically in a concentration-dependent manner, reaching 42%, 48%, 55%, and 65% at 0.25, 0.50, 0.75, and 1.00 mM H_2_O_2_, respectively (Figure 1c).

This synergistic effect can be rationalized by the chemical nature of monascin and the photochemistry of H_2_O_2_. Monascin possesses an azaphilone skeleton with an extended conjugated pyranone chromophore that absorbs light efficiently (λmax = 385 nm) [21]. While this conjugated system is susceptible to photoexcitation, it exhibits intrinsic resistance to mild oxidants such as H_2_O_2_ under dark conditions. The reaction between H_2_O_2_ and the chromophore is kinetically unfavorable because the azaphilone core lacks strongly electron-withdrawing or electron-donating substituents that would facilitate electrophilic attack, and the steric hindrance from side chains further limits oxidant accessibility. Consequently, direct oxidation by H_2_O_2_ alone proceeds slowly, as reflected by the low degradation rates observed.

Upon irradiation, however, H_2_O_2_ undergoes photolytic cleavage of its O–O bond (H_2_O_2_ + hν → 2·OH), generating hydroxyl radicals (·OH), among the most reactive oxidants known [22]. These radicals react with the conjugated chromophore at near-diffusion-limited rates, leading to rapid and extensive degradation. The pronounced synergistic effect observed under combined light and H_2_O_2_ exposure is therefore attributed to the photochemical generation of ·OH radicals, which efficiently attack the chromophore, whereas H_2_O_2_ alone lacks sufficient oxidative power to cause significant degradation. This interpretation is further supported by the ESR detection of ·OH radicals under irradiation (see Section 3.2) and by the protective effects of radical scavengers (Section 3.4 and Section 3.5).

### 3.2. ·OH Radicals Generated During Irradiation

**Figure 2:**
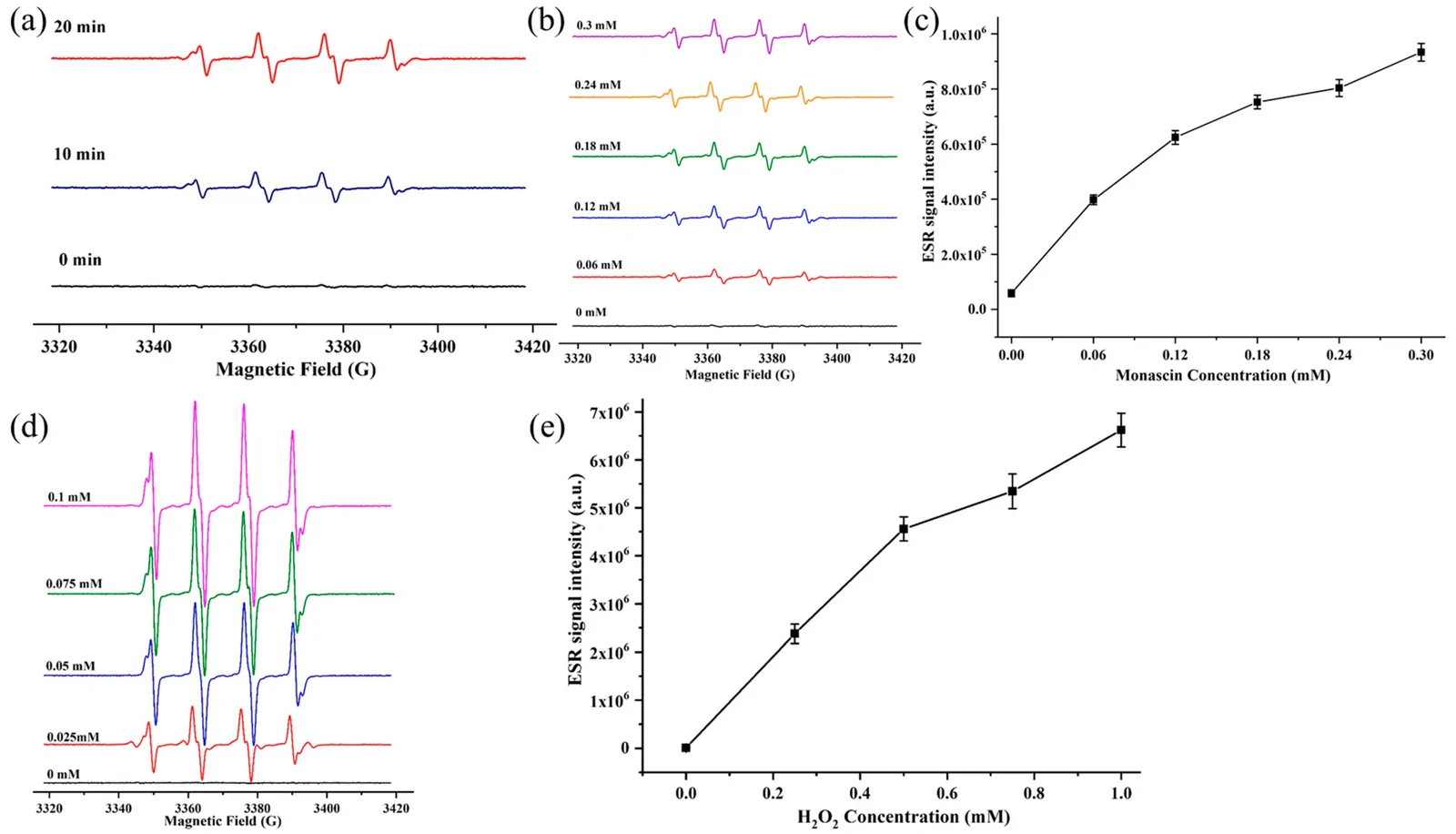
Free radical generation from monascin and H_2_O_2_ under simulated solar light irradiation. (**a**) Time-dependent ESR spectra of radicals produced from 0.3 mM monascin under simulated solar light irradiation. (**b**) ESR spectra of free radicals generated by different concentrations of monascin after 10 min irradiation. (**c**) ESR signal intensity of free radicals from varying monascin concentrations irradiated for 10 min. (**d**) ESR spectra of radicals generated by different concentrations (0.25–1.00 mM) of H_2_O_2_ after 10 min irradiation. (**e**) ESR signal intensity corresponding to (**d**). Data are presented as mean ± SD (*n* = 3).

To investigate free radical generation during monascin photo-degradation, electron spin resonance (ESR) spectroscopy coupled with BMPO spin-trapping was employed. ESR measurements were performed as described in Section 2.8, using samples prepared according to the dilution procedure in Section 2.2. The characteristic four-line ESR spectrum with a 1:2:2:1 intensity ratio is diagnostic of the BMPO/·OH adduct, unequivocally confirming hydroxyl radical generation (Figure 2).

As shown in Figure 2a, irradiation of monascin alone produced a time-dependent increase in ESR signal intensity, with 20 min exposure generating significantly stronger signals than 10 min. The 1:2:2:1 quartet spectrum confirms that ·OH radicals are generated during monascin photo-degradation. Furthermore, the ESR signal intensity increased progressively with monascin concentration (0.06–0.3 mM) after 10 min irradiation (Figure 2b,c), with intensities ranging from 3.98 × 10^6^ a.u. to 9.33 × 10^6^ a.u. This concentration-dependent enhancement indicates that monascin acts as the primary photo sensitizer; upon photon absorption, monascin molecules transition to excited states and facilitate electron transfer processes that yield ·OH radicals. These radicals then attack the pigment chromophore, driving photo-degradation and discoloration, consistent with previous reports on photoexcitation-induced radical formation in related pigments [22,23].

To confirm that ·OH radicals can be generated from H_2_O_2_ photolysis independently of monascin, control experiments were performed in which H_2_O_2_ solutions (0.25–1.00 mM in 70% acetonitrile) were irradiated under identical conditions without monascin. As shown in Figure 2d,e, a clear concentration-dependent increase in the BMPO/·OH signal was observed, confirming that photolytic cleavage of H_2_O_2_ produces ·OH radicals even in the absence of monascin. These control experiments establish that the synergistic degradation observed under combined light and H_2_O_2_ exposure (Section 3.1) is attributable to ·OH radicals, which subsequently attack the monascin chromophore [24,25]. Notably, while H_2_O_2_ is a relatively selective oxidant, ·OH reacts unselectively with most organic molecules at near-diffusion-limited rates, leading to rapid ring opening, fragmentation, and bleaching of the conjugated chromophore [26]. The complementary evidence from Figure 2a–c (monascin-derived radical generation) and Figure 2d,e (H_2_O_2_-derived radical generation) collectively demonstrates that both the pigment itself and exogenous H_2_O_2_ can serve as sources of ·OH under irradiation, with the latter producing substantially higher radical yields and correspondingly greater degradation rates. These findings provide the mechanistic basis for the enzymatic and antioxidant intervention strategies examined in subsequent sections.

### 3.3. Effect of Deoxygenation on Monascin Photo-Stability

**Figure 3:**
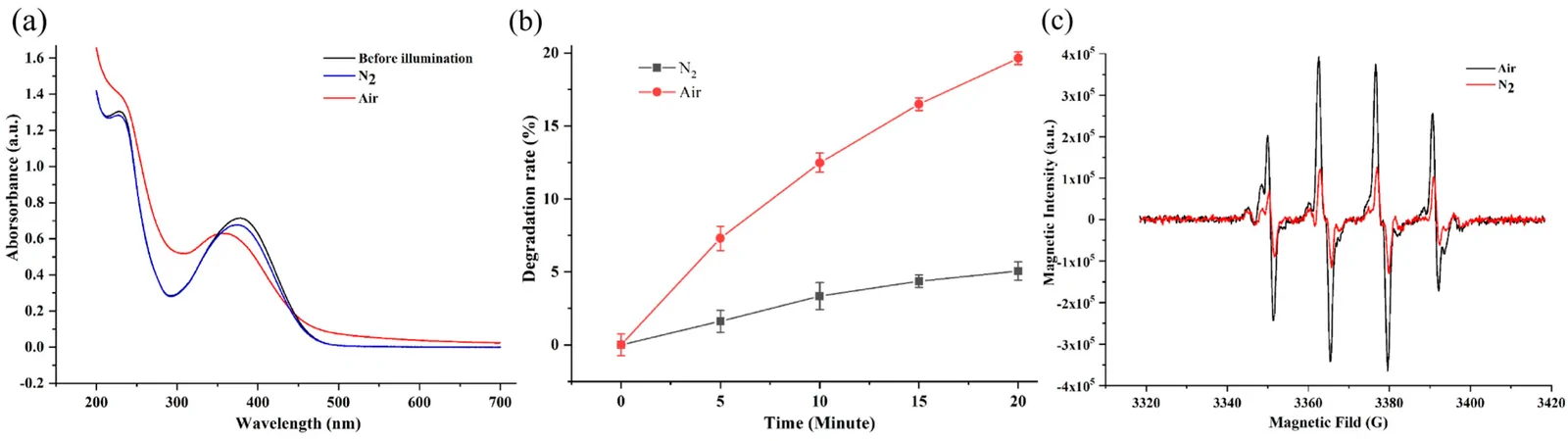
Effect of deoxygenation on the light stability and free radical generation of 0.18 mM monascin. (**a**) UV-vis absorption spectra of monascin before (black) and after 20 min irradiation in deoxygenated (blue) and non-deoxygenated (red) solutions. (**b**) Degradation rates of monascin under deoxygenated and non-deoxygenated conditions after 20 min irradiation. (**c**) ESR signal intensities of free radicals generated after 10 min irradiation in deoxygenated versus non-deoxygenated solutions. Data are presented as mean ± SD (*n* = 3).

Environmental factors, particularly the concentration of dissolved oxygen, play a critical role in the photo-stability of pigments. To assess the role of dissolved oxygen, the photo-stability and radical generation of monascin under illumination in non-deoxygenated solutions and deoxygenated solutions was investigated (Figure 3).

As shown in Figure 3a, irradiation of the non-deoxygenated solution for 20 min caused a pronounced decrease in the characteristic absorption peak at 385 nm, accompanied by a blue shift, with a 19.65% reduction in absorbance. In contrast, under deoxygenated conditions, the spectral changes were substantially attenuated, and degradation was markedly reduced to only 5% (Figure 3b). These results indicate that the presence of dissolved oxygen significantly accelerates pigment fading.

Molecular oxygen is known to interact with photoexcited chromophores to generate reactive oxygen species (ROS), including peroxides, hydroxyl radicals, superoxide anions, and singlet oxygen [24,25]. These species readily attack susceptible functional groups in pigment molecules, leading to irreversible structural damage. Consistent with this mechanism, deoxygenation by nitrogen purging reduced the ESR signal intensity of radicals generated after 10 min irradiation by 68% compared to the non-deoxygenated control (Figure 3c). This substantial reduction in radical yield directly correlates with the observed improvement in photo-stability. Collectively, these findings demonstrate that dissolved oxygen amplifies radical formation and accelerates monascin photo-degradation. The observed 68% reduction in radical generation and the decline in degradation from 19.65% to 5% upon deoxygenation indicate that oxygen exclusion is an effective strategy for enhancing monascin photo-stability. We acknowledge that the precise quantitative relationship between dissolved oxygen concentration and degradation rate was not established in this study, as only two conditions (ambient air and nitrogen-purged) were compared. Nonetheless, these results clearly establish that oxygen removal significantly suppresses radical-mediated degradation [27].

### 3.4. Effect of CAT and SOD on the Light Stability of Monascin

**Figure 4:**
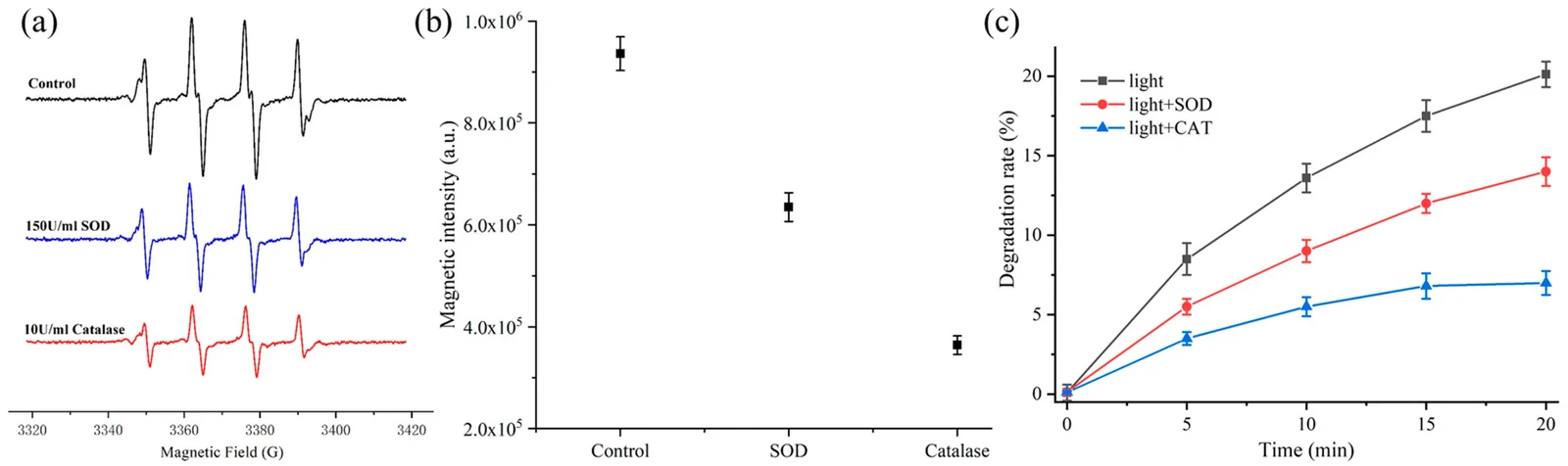
Effects of CAT and SOD on free radical generation and photo-stability of 0.3 mM monascin. (**a**) ESR spectra of free radicals generated from 0.3 mM monascin solution after 10 min irradiation in the presence of 150 U/mL SOD or 10 U/mL CAT. (**b**) ESR signal intensity corresponding to (**a**). (**c**) Degradation kinetics of monascin over 20 min irradiation with CAT or SOD. Data are presented as mean ± SD (*n* = 3).

Given the potential involvement of reactive oxygen species (ROS) in the photo-degradation process, we investigated whether hydrogen peroxide (H_2_O_2_) and superoxide anions (O_2_·^−^) are generated during illumination and contribute to monascin photo-degradation. To elucidate the specific roles of these species, the effects of catalase (CAT), which specifically decomposes H_2_O_2_, and superoxide dismutase (SOD), which dismutates O_2_·^−^ to H_2_O_2_ and O_2_, were investigated. These enzymes serve as selective probes to distinguish whether the observed photo-degradation is primarily mediated by H_2_O_2_-derived ·OH or by superoxide-dependent pathways (Figure 4).

As shown in Figure 4a,b, the addition of either CAT or SOD significantly suppressed radical formation compared to the control. SOD (150 U/mL) reduced the ESR signal intensity by approximately 32% after 10 min irradiation, while CAT at a 15-fold lower concentration (10 U/mL) exhibited a substantially stronger inhibitory effect, reducing the radical signal by 61%. These results provide direct spectroscopic evidence that both H_2_O_2_ and O_2_^−^· are generated during photo-degradation and participate in the radical cascade, while also revealing the predominant role of H_2_O_2_-derived species in this process.

Consistent with the ESR observations, the degradation kinetics (Figure 4c) mirrored the radical suppression patterns. After 20 min irradiation, the degradation rate of monascin in the absence of any scavenger reached 20%. SOD (150 U/mL) reduced this to 14%, whereas CAT (10 U/mL) provided superior protection, limiting degradation to only 7%. This close correspondence between radical suppression and degradation reduction confirms that radical scavenging directly translates into photo-protection.

The superior protective effect of catalase can be attributed to the central role of H_2_O_2_ as the direct precursor to ·OH. Under irradiation, H_2_O_2_ undergoes homolytic fission to generate ·OH, a non-selective and highly reactive oxidant capable of attacking the monascin chromophore at near-diffusion-limited rates. By rapidly decomposing H_2_O_2_ into H_2_O and O_2_, CAT intercepts the degradation pathway at its most critical juncture, effectively starving the system of the feedstock for ·OH production and terminating the radical chain reaction at its source [28].

In contrast, SOD acts on a more upstream pathway. O_2_·^−^ is likely generated via electron transfer from the excited triplet state of monascin to molecular oxygen [29]. However, O_2_·^−^ itself is a relatively mild and selective oxidant compared to ·OH. Its primary role in accelerating degradation is indirect: it serves as a precursor to H_2_O_2_ and can participate in metal-catalyzed reactions to regenerate ·OH [30]. Notably, SOD catalysis generates H_2_O_2_ as a product. If this H_2_O_2_ is not subsequently removed, it remains available for photolytic conversion to ·OH. This explains why even a high concentration of SOD (150 U/mL) achieved only partial inhibition of radical formation (32%) and a modest reduction in degradation rate (from 20% to 14%).

### 3.5. Effect of Ascorbic Acid and α-Lipoic Acid on Monascin Photo-Stability

**Figure 5:**
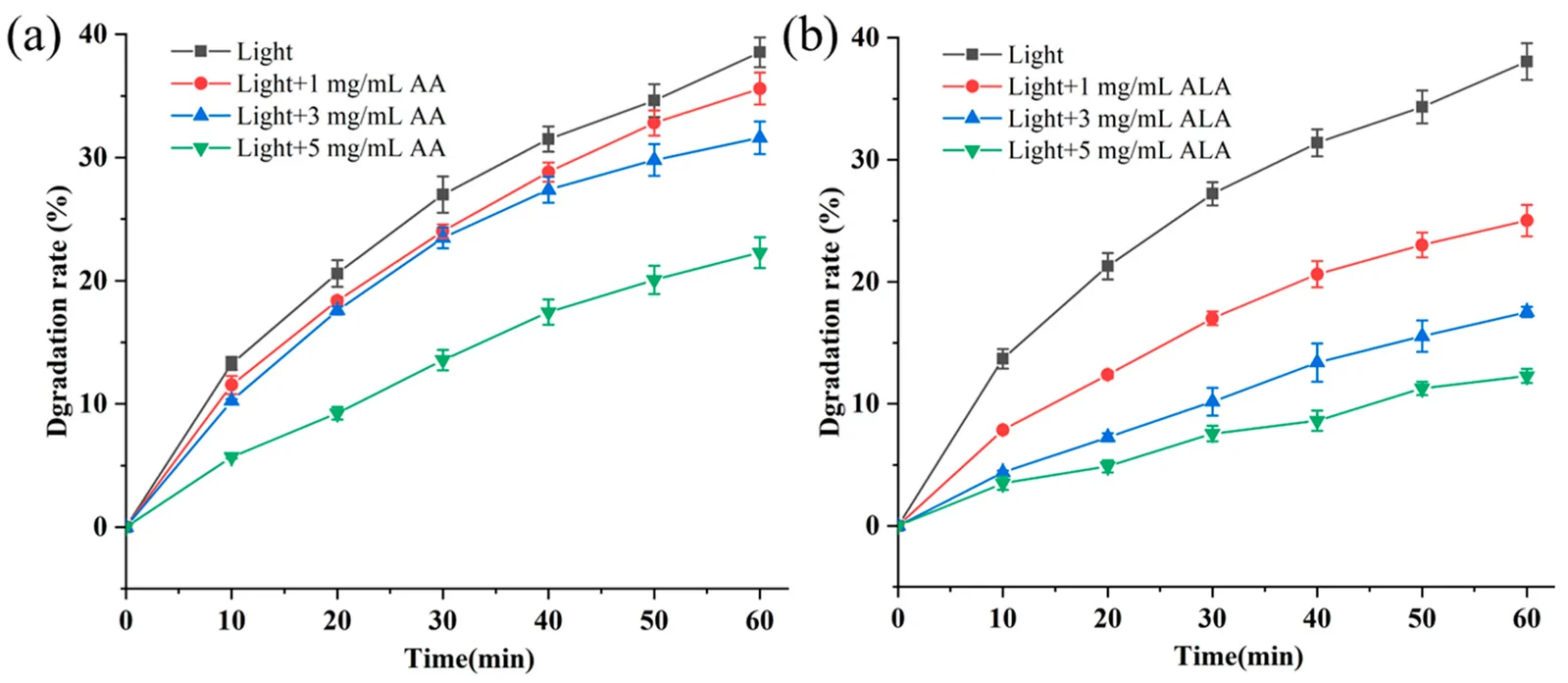
Effect of AA and ALA on monascin photo-stability. (**a**) Degradation kinetics of monascin with AA at 1, 3, and 5 mg/mL. (**b**) Degradation kinetics of monascin with ALA at 1, 3, and 5 mg/mL. Data are presented as mean ± SD (*n* = 3).

Many naturally occurring antioxidants are known to scavenge reactive oxygen species (ROS), including hydroxyl radicals (·OH), superoxide anions (O_2_·^−^), and singlet oxygen (^1^O_2_), thereby exerting protective effects on the photo-stability of various pigments. To further explore potential strategies for enhancing monascin photo-stability, we investigated the effects of two representative antioxidants, ascorbic acid (AA) and α-lipoic acid (ALA), on monascin degradation under simulated solar light irradiation (Figure 5).

As shown in Figure 5, both AA and ALA reduced the photo-degradation rate of monascin in a concentration-dependent manner. After 60 min irradiation, monascin alone exhibited 38.50% degradation. The addition of 5 mg/mL AA reduced the degradation rate to 22.29% (42% reduction), while the same concentration of ALA provided significantly greater protection, limiting degradation to only 12.28% (68% reduction). This protective effect diminished with decreasing antioxidant concentration; at 1 mg/mL, the degradation rates after 60 min of illumination were 35.60% for AA and 25.02% for ALA. This concentration-dependent trend indicates that at higher concentrations, both antioxidants compete effectively with monascin for ROS scavenging. At lower concentrations, competition becomes less favorable, allowing more ROS to attack the chromophore. The superior performance of ALA compared to AA at both concentrations provides strong supporting evidence that ·OH is the key mediator of photo-degradation, consistent with our ESR and enzymatic results.

This significant difference in efficacy between ALA and AA can be attributed to several factors related to their distinct physicochemical and antioxidant properties. First, ALA possesses a potent redox couple capable of scavenging a broad range of ROS, including singlet oxygen, ·OH, and O_2_^−^·, and (^1^O_2_) [31,32], whereas AA is primarily effective against aqueous-phase radicals and has limited activity against ^1^O_2_ [33,34]. Notably, the AA solutions used in this study contained EDTA to chelate trace metal ions, and no exogenous H_2_O_2_ was introduced, effectively excluding Fenton chemistry under our experimental conditions. Second, ALA is amphiphilic, allowing it to partition at interfaces and interact more efficiently with the relatively hydrophobic monascin chromophore in the 70% acetonitrile system. AA is highly hydrophilic and primarily localized in the aqueous phase. Third, ALA can chelate transition metal ions (e.g., Fe^2+^, Cu^2+^), potentially suppressing Fenton chemistry that converts H_2_O_2_ into ·OH [35,36]. While direct quenching of the excited triplet state of monascin by ALA cannot be excluded, the observed protective effect is primarily attributed to its broad-spectrum ROS scavenging capacity and metal-chelating activity.

## 4. Conclusions

This study demonstrates that hydroxyl radical (·OH) generation constitutes a key mechanism underlying monascin photo-degradation. Under simulated sunlight irradiation (100 mW/cm^2^), monascin undergoes photoexcitation and electron transfer, leading to the production of reactive oxygen species (ROS), including superoxide anions (O_2_^−^·) and hydrogen peroxide (H_2_O_2_), with H_2_O_2_ serving as the critical precursor to ·OH via photolytic cleavage. Dissolved oxygen markedly amplifies this radical cascade, as evidenced by the 68% reduction in radical yield and the decline in degradation rate from 19.65% to 5% upon deoxygenation.

The differential effects of specific enzymatic scavengers establish H_2_O_2_ as a central regulatory target. Notably, catalase, even at a 15-fold lower concentration than superoxide dismutase, achieved superior protection by decomposing H_2_O_2_ and thereby terminating ·OH generation at its source. This positions H_2_O_2_ scavenging as a more effective strategy than intervening at the upstream O_2_^−^· level. Among the natural antioxidants evaluated, α-lipoic acid exhibited markedly superior photo-protective efficacy compared to ascorbic acid, reducing monascin degradation by 68% at 5 mg/mL. This enhanced protection is attributed to ALA’s broad-spectrum ROS-scavenging capacity (including direct ·OH), its amphiphilic nature enabling favorable partitioning near the hydrophobic monascin chromophore, and its potential to suppress Fenton chemistry via transition metal chelation. Importantly, the primary purpose of comparing AA and ALA was to validate the central role of ·OH in monascin photofading, with the superior performance of ALA providing strong supporting evidence for this mechanism.

Collectively, these findings establish that photo-degradation of monascin proceeds through a radical-mediated mechanism with H_2_O_2_-derived ·OH as the primary destructive species. Effective photo-stabilization strategies should therefore prioritize oxygen exclusion, H_2_O_2_ decomposition, and employment of multifunctional antioxidants such as α-lipoic acid. In addition to the chemical strategies identified here, physical shielding approaches (e.g., UV absorbers or encapsulation) merit further investigation as complementary means to enhance monascin photo-stability in practical applications.

Several limitations of this study should be acknowledged. First, all experiments were conducted in a 70% acetonitrile model system, which does not fully replicate the complexity of real food matrices. Factors such as pH, competitive reactions with other food components, and light penetration may influence the efficacy of the proposed strategies in practices. Second, while we have identified hydroxyl radicals as the primary reactive species driving monascin photo-degradation and have demonstrated the protective effects of enzymatic scavengers and antioxidants, we did not perform comprehensive structural elucidation of the photo-degradation products, which would be required to chart a complete chemical degradation pathway. Third, the detailed mechanism of α-lipoic acid (ALA) protection, particularly whether it involves quenching of the excited triplet state of monascin, remains to be resolved. Fourth, the relationship between color fading and the loss of biological activities (e.g., anti-diabetic or anti-inflammatory effects) has not been investigated, which is critical for functional applications. Finally, the scalability of the proposed strategies, such as deoxygenation or enzyme addition, to food processing conditions requires further evaluation. These limitations represent important directions for our ongoing and future research.

## Data Availability

The data that support the findings of this study are available from the corresponding author upon reasonable request.
